# The Prevalence of Sarcopenia in Chinese Older Adults: Meta-Analysis and Meta-Regression

**DOI:** 10.3390/nu13051441

**Published:** 2021-04-24

**Authors:** Zi Chen, Wei-Ying Li, Mandy Ho, Pui-Hing Chau

**Affiliations:** School of Nursing, The University of Hong Kong, Hong Kong, China; u1chenzi@connect.hku.hk (Z.C.); u3003886@connect.hku.hk (W.-Y.L.); mandyho1@hku.hk (M.H.)

**Keywords:** sarcopenia, prevalence, nutrition, physical activity, meta-analysis, meta-regression

## Abstract

Sarcopenia, with risk factors such as poor nutrition and physical inactivity, is becoming prevalent among the older population. The aims of this study were (i) to systematically review the existing data on sarcopenia prevalence in the older Chinese population, (ii) to generate pooled estimates of the sex-specific prevalence among different populations, and (iii) to identify the factors associated with the heterogeneity in the estimates across studies. A search was conducted in seven databases for studies that reported the prevalence of sarcopenia in Chinese older adults, aged 60 years and over, published through April 2020. We then performed a meta-analysis to estimate the pooled prevalence, and investigated the factors associated with the variation in the prevalence across the studies using meta-regression. A total of 58 studies were included in this review. Compared with community-dwelling Chinese older adults (men: 12.9%, 95% CI: 10.7–15.1%; women: 11.2%, 95% CI: 8.9–13.4%), the pooled prevalence of sarcopenia in older adults from hospitals (men: 29.7%, 95% CI:18.4–41.1%; women: 23.0%, 95% CI:17.1–28.8%) and nursing homes (men: 26.3%, 95% CI: 19.1 to 33.4%; women: 33.7%, 95% CI: 27.2 to 40.1%) was higher. The multivariable meta-regression quantified the difference of the prevalence estimates in different populations, muscle mass assessments, and areas. This study yielded pooled estimates of sarcopenia prevalence in Chinese older adults not only from communities, but also from clinical settings and nursing homes. This study added knowledge to the current epidemiology literature about sarcopenia in older Chinese populations, and could provide background information for future preventive strategies, such as nutrition and physical activity interventions, tailored to the growing older population.

## 1. Introduction

The speed of population ageing is accelerating globally. According to statistics from the World Health Organization (WHO), by 2050, the proportion of individuals over the age of 60 is expected almost to double (22%) compared with 12% in 2015 [1]. China is already witnessing this demographic trend; thus, the country’s National Bureau of Statistics has predicted that the number of adults aged 65 years and above will increase from 166.58 million (11.9% of the total population) in 2018 to 366 million in 2050 [2].

Advancing age is marked by a series of physiological changes in body composition, including the decrease in skeletal muscle mass and increase in fat mass [3,4]. Sarcopenia is the age-related decline in skeletal muscle mass and function characterized by the loss of muscle strength and physical performance [5]. Accordingly, understanding more about its etiology and risk factors is of great interest. The onset and progression of sarcopenia can be attributed to numerous factors including physical inactivity and poor nutrition [6]. Therefore, most non-pharmacological interventions about sarcopenia mainly target these two modifiable factors. An umbrella review concluded that exercise training, especially resistance training, had a significant effect on the improvement of muscle mass, muscle strength, and physical performance [7]. Bloom et al. systematically reviewed observational evidence regarding the relationship of diet quality and sarcopenia, and concluded that a higher quality diet was associated with better physical performance among older adults [8]. Furthermore, a narrative review conducted by Tessier et al. examined the observational and interventional evidence regarding the association between some specific nutrients and sarcopenic components [9]. They found that some nutrients such as proteins, leucine, vitamin D, and n-3 polyunsaturated fatty acids (n-3 PUFAs) might have a protective impact on muscle health among older adults [9]. Previous research has associated sarcopenia with such adverse health outcomes as fractures, falls, functional decline, hospitalization, and even increased mortality [10,11]. Hence, early screening and identifying sarcopenia among older populations should be at the forefront of timely diet and/or exercise interventions for sarcopenia prevention and treatment.

Sarcopenia is diagnosed based on low muscle mass, low muscle strength, and diminished physical performance. However, no standard and unique diagnosis criteria for sarcopenia have yet been established. At present, several international groups, such as the European Working Group on Sarcopenia in Older People (EWGSOP), Asia Working Group for Sarcopenia (AWGS), International Working Group on Sarcopenia (IWGS), Foundation for the National Institutes of Health (FNIH) Sarcopenia Project, have provided their own diagnostic criteria for sarcopenia [12,13,14]. Estimates of sarcopenia prevalence are, in turn, dependent on the diagnostic criteria used to define it. For example, a longitudinal multi-center cohort research found the prevalence of sarcopenia among community-dwelling older adults ranged from 3.3% to 17.5% depending on the diagnostic criteria used (specifically, AWGS: 9.1%, EWGSOP: 17.5%, IWGS: 16.1%, and FNIH: 3.3%) [15]. Assessment of muscle mass is an essential part of sarcopenia diagnosis. Dual-energy X-ray absorptiometry (DXA) and bioelectrical impedance analysis (BIA) are both recommended to assess muscle mass in research and practice [12]. Therefore, estimates of the prevalence may also depend on different assessment approaches. For example, Beaudart et al. [16] compared the prevalence of sarcopenia (EWGSOP criterion) using different muscle mass assessments (DXA and BIA) among older adults over 65 years, and found the prevalence was lower when using the BIA technique (BIA vs. DXA: 12.8% vs. 21%). Likewise, there is evidence that the methods used to measure muscle strength and physical performance may yield inconsistent estimates of the prevalence of sarcopenia [17]. Furthermore, estimates of prevalence also varied across populations and areas [18,19,20,21,22,23]. Previous evidence revealed that hospitalized older adults and nursing-home residents had higher prevalence of sarcopenia compared with community-dwelling older residents [18]. Community-dwelling Chinese older adults residing outside mainland China (i.e., Hong Kong and Taiwan) showed a lower prevalence rate of sarcopenia than counterparts from the mainland [24].

At present, two systematic reviews have pooled the estimate of sarcopenia prevalence in community-dwelling Chinese older adults (17% and 11%, respectively) [24,25]. However, few systematic reviews have pooled the prevalence for Chinese older adults in other settings, such as clinical settings and nursing homes. Furthermore, no study has systematically investigated the factors contributing to the heterogeneity in the estimates of sarcopenia prevalence through meta-regression. Therefore, we conducted a meta-analysis and meta-regression to shed light on the prevalence of sarcopenia in Chinese older adults not only from communities, but also from clinical settings and nursing homes, and to explain the heterogeneity in sarcopenia prevalence across studies.

Information about the prevalence of sarcopenia is the first step to develop preventive routines or health services tailored to the growing older population. Meta-analysis and meta-regression of prevalence data are increasingly important for policy making and implementation of preventive measures in situations for which inconsistent prevalence estimates have been reported in the literature. This study could help policy makers and health practitioners make evidence-based decisions targeting the health issues of sarcopenia.

## 2. Methods

This review was conducted in accordance with the Preferred Reporting Items for Systematic Reviews and Meta Analyses (PRISMA) statement [26].

### 2.1. Search Methods

Two researchers (Z.C. and W.Y.L.) independently searched the following online electronic databases: PubMed, Cochrane Library, Embase (Ovid), CINAHL, Web of Science Core Collection, CNKI, and Wanfang (accessed on 15 April 2020); the latter two being Chinese. The search period was restricted from the earliest records to 15 April, 2020. Key search terms were “sarcopenia”, “epidemiology”, “prevalence”, “aged”, “elderly”, “older”, “China”, and “Chinese”. Logical operators (i.e., AND, OR) were used to shape the search strategy. Detailed search strategies for PubMed and Wanfang are presented in the Supplementary Materials as examples (S1: Search strategy). Relevant studies were identified through manual searches in the reference list of eligible studies.

Inclusion criteria were as followed: (1) the prevalence of sarcopenia was reported or could be calculated; (2) participants were of Chinese ethnicity; (3) the age was 60 years and over; (4) participants were recruited from the community, clinical settings, or nursing homes; (5) EWGOSP, AWGS, IWGS or FINH definitions of sarcopenia were adopted; (6) muscle mass was measured with DXA or BIA; (7) primary research irrespective of design. Exclusion criteria were (1) studies published as reviews, letters to editors, conference abstracts, expert opinions, case reports; (2) studies published in languages other than English and Chinese.

First, duplicate records were identified and removed. Then, titles and abstracts were screened to remove studies irrelevant to the research questions. Next, full-text articles were retrieved and reviewed for eligibility according to the selection criteria. When multiple papers came from the same dataset, we chose the one with the largest sample size which might be closer to the original cohort in order to avoid those using a particular subset of the whole dataset. The above process was performed independently by the two researchers (Z.C. and W.-Y.L.). Any discrepancies were adjudicated by a third researcher (P.-H.C.).

### 2.2. Data Extraction

The following details were extracted from each of the eligible studies: the year of publication, country or area in which the data were collected, research design, sample size, settings where participants were recruited, diagnostic criteria for sarcopenia, measurement of muscle mass, muscle strength, and physical performance and prevalence of sarcopenia. For cohort or intervention studies, only baseline data were extracted. For studies using more than one diagnostic criterion to define sarcopenia, all estimates of prevalence were extracted.

### 2.3. Critical Appraisal

Two researchers (Z.C. and W.Y.L.) independently assessed the quality of included studies using a validated tool developed by Hoy et al., which was tailored to assess the risk of bias for prevalence studies with different types of design [27]. The tool evaluates the risk of bias through 10 items. The first four items mainly focus on external validity, which involve the representativeness of the target population and the sampling frame, selection of the sample, and non-response bias. The remaining items address the issue of internal validity, which involves the use of proxy respondent or not, acceptable case definition, validated measurement, consistent mode of data collection, the length of the shortest prevalence period, and correct calculation of prevalence. Each item was rated as “low risk” or “high risk”. When information in the article was not sufficient for judgement, that item was rated as “high risk”. Following previous literature, the study was considered to have a low risk of bias when 9 or 10 items were rated “low risk”; a moderate risk of bias when 6 to 8 items were rated “low risk”; and a high risk of bias when 5 or less items were rated “low risk” [28,29].

### 2.4. Statistical Analysis

The prevalence of sarcopenia varied in different genders and populations recruited from different settings [18]. Accordingly, in this study, the pooled prevalence was obtained separately for each gender and for the population from each setting. For studies which recruited participants from mixed settings, only those which reported the prevalence for each setting were included in the meta-analysis. While more than one estimate was extracted from studies which used more than one diagnostic criterion to define sarcopenia, only the one that was more frequently used in other eligible studies was used for calculation of the pooled estimate.

Random-effect models take into account the possibility that the parameters for the population may vary among studies [30]. Therefore, because of the variations in populations, diagnosis criteria, muscle mass assessments, etc., among the eligible studies, we chose a random-effect model to calculate the pooled prevalence. The *metaprop* command in Stata 15.0 (Stata Corp, College Station, TX, USA) was used to obtain pooled estimates of prevalence [31,32].

We assessed the heterogeneity in the estimates across studies using Cochran’s Q test, with a *p*-value < 0.10 indicating heterogeneity [33]. We quantified the heterogeneity using I-square, with I^2^ statistics of 25%, 50%, and 75% set as the cut-offs for low, moderate, and high heterogeneity, respectively [33]. If heterogeneity existed, random-effect meta-regression was conducted to explore the potential source of variability in prevalence estimates across studies. Meta-regression can explore the effects of multiple study characteristics on the variance of pooled estimates simultaneously [30]. For this study, we performed multivariable meta-regression (using the *metareg* command) to model the adjusted association between multiple explanatory variables and prevalence estimates. Based on previous evidence, we considered the following study characteristics to be potential sources of heterogeneity: populations from different settings, diagnostic criteria for sarcopenia, assessment of muscle mass, muscle strength and physical performance, and the area of study. In order to have a sufficient power, meta-regression was only performed for covariates reported in at least ten studies [33].

Publication bias refers to the phenomenon that studies with significant results are more likely to get published than those with negative results, which can result in systematic differences between published and unpublished studies [34]. In the case of observational studies that report prevalence, however, there are no positive or negative results, and no well-established method is recommended to test for this bias in meta-analysis of prevalence studies. Therefore, in this study, we did not check for publication bias.

## 3. Results

### 3.1. Search Outcomes

**Figure 1:**
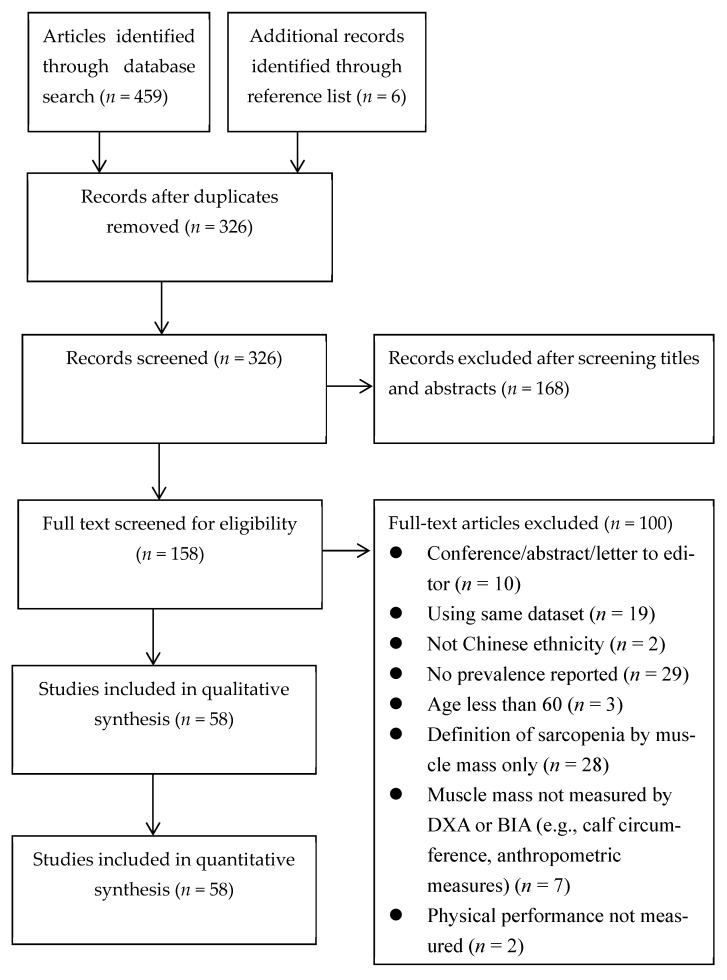
PRISMA flowchart.

We identified 459 records from databases and 6 records from the reference lists. After removing 139 duplicated records, we screened the titles and abstracts of the remaining 326 records, and removed another 168 irrelevant records, leaving 158 studies. Further reviewing the full texts of these studies for eligibility, we excluded another 100 for the reasons specified in Figure 1. We carried out the quantitative synthesis (i.e., meta-analysis and meta-regression) on the remaining 58 records.

The characteristics of the included studies are summarized in Table 1. Fifty-one (87.9%) studies were classified as having a moderate risk of bias and seven (12.1%) were classified as having a low risk of bias. None of the included studies recruited the nationwide representative sample. Twelve studies adopted random sampling [19,20,35,36,37,38,39,40,41,42,43,44]. One study was open to the non-response bias [35], Each study collected data of sarcopenia components directly from subjects and had a consistent mode of data collection. All included studies had acceptable case definition and correct calculation of prevalence. Details of the critical appraisal are presented in Supplementary Materials (Table S2).

### 3.2. Prevalence in Older Men

In the 46 studies that reported the prevalence of sarcopenia in older men, the overall prevalence was 18% (95% CI: 15.7 to 20.4%, I^2^ = 95.2%). For participants from different settings, the pooled prevalence rates were 12.9% (95% CI: 10.7 to 15.1%, I^2^ = 93.7%) in community-dwelling older men (*n* = 27), 29.7% (95% CI: 18.4 to 41.1%, I^2^ = 95.5%) for hospitalized older men (*n* = 9), and 26.3% (95% CI: 19.1 to 33.4%, I^2^ = 83.7%) in nursing-home residents (*n* = 5). We did not pool the prevalence for outpatients due to an insufficient number of studies (*n* = 2). Substantial heterogeneity in prevalence estimates was found across the studies, with all of the I-square values being greater than 80%.

### 3.3. Prevalence in Older Women

In the 44 studies that reported the prevalence of sarcopenia in older women, the overall prevalence was 16.4% (95% CI: 14.1 to 18.8%, I^2^ = 97.3%). For participants from different settings, the prevalence was 11.2% (95% CI: 8.9 to 13.4%, I^2^ = 97.1%) in community-dwelling older women, (*n* = 27), 23.0% (95% CI: 17.1 to 28.8%, I^2^ = 80.9%) for hospitalized older women (*n* = 9), and 33.7% (95% CI: 27.2 to 40.1%, I^2^ =78.4%) for those from nursing homes (*n* = 4). Because only one study reported the prevalence for female outpatients, we did not perform meta-analysis for outpatients. Similarly, considerable heterogeneity was found across studies.

### 3.4. Meta-Regression Analysis

We performed a multivariable meta-regression to explore the potential sources of the considerable heterogeneity in the estimates of sarcopenia prevalence across studies. The results of the regression indicated that, irrespective of gender, the hospitalized patients and nursing-home residents had a higher prevalence of sarcopenia than community-dwelling older adults. For older men, the prevalence rate was lower when muscle mass was assessed using the BIA method rather than the DXA method. Furthermore, those from mainland China appeared to have a higher prevalence of sarcopenia compared with those from Hong Kong or Taiwan. For older women, only the participants from different settings showed a significant association with the prevalence of sarcopenia (Table 2).

## 4. Discussion

This meta-analysis provided a comprehensive picture of the prevalence of sarcopenia in Chinese older adults. It filled the knowledge gap by extending the existing reviews of sarcopenia prevalence in Chinese older adults from communities to other settings (clinical settings and nursing homes) as well. We also systematically investigated factors that contributed to the significant heterogeneity in pooled prevalence estimates using multivariable meta-regression. Our findings contribute to the epidemiological literature on sarcopenia in Chinese older adults, and can provide a starting point for future research and efforts in sarcopenia prevention among Chinese populations.

For community-dwelling Chinese older adults, we obtained similar estimates of prevalence (12.9% in men vs. 11.2% in women) compared with previous reports (11% in men vs. 10% in women; 14% in men vs. 9% in women) [24,25]. When compared with the prevalence in Europeans (13% in men vs. 14% in women), our estimates were comparable with them [18]. However, Papadopoulou et al. [18] found non-Asian groups seemed more prone to sarcopenia than Asian groups. The difference in ethnic characteristics, body size, and dietary regimes etc., between Asians and non-Asians might be possible reasons for the potential disparity in the sarcopenia prevalence. As for gender difference, our result was similar to the previous evidence that higher prevalence of sarcopenia was shown in Chinese community-dwelling older males than females [15,22,25]. However, in western countries, the evidence of gender difference was controversial depending on the EWGSOP cut-off values applied [13,87]. For hospitalized older adults, we found the prevalence to be 29.7% in men and 23.0% in women. To our knowledge, this is the first systematic review to pool the prevalence of sarcopenia for Chinese older adults who are hospitalized. Our findings revealed a relatively higher prevalence in this group compared with that from communities. Compared with the pooled estimates of hospitalized older adults mainly from Europe (23% in men and 24% in women), our findings were higher in hospitalized men and comparable in hospitalized women [18]. For outpatients, considering that Cochran’s Q is not very informative and tends to be biased when the number of studies is small, we did not perform meta-analysis for this group [88]. For those residing in nursing homes, similarly, no meta-analysis synthesized the prevalence of sarcopenia in this Chinese group before. Shen et al. [28] conducted a systematic review pooling the prevalence of sarcopenia in nursing-home populations from 16 studies. However, they only included one original study that targeted the Chinese population [28]. Compared with Shen et al.’s results (43% in men vs. 46% in women), we obtained lower pooled estimates (26.3% in men vs. 33.7% in women) [28]. Nevertheless, the above evidence suggested the high risk of prevalent sarcopenia in nursing-home residents. Institutionalized populations may be more prone to malnutrition [89,90]. A recent cross-sectional study found that malnutrition and physical frailty were highly prevalent among institutionalized older residents and malnutrition was associated with an increased risk of physical frailty among institutionalized Chinese older adults [89]. Hence, nutrition and exercise intervention should be promoted to the institutionalized population to prevent or reverse muscle function for this population.

Multivariable meta-regression indicated that the populations being from different settings was a significant factor contributing to the variation in the pooled prevalence estimates for both genders. Compared with community-dwelling older residents, those who were hospitalized were at 1.69 to 2.10 times the risk of prevalent sarcopenia, and nursing-home residents had 2.50 to 2.73 times the risk. The much higher prevalence rate of sarcopenia in older adults from hospitals and nursing homes was also reported by Papadopoulou and colleagues [18]. However, while Papadopoulou and colleagues reported variation in the pooled prevalence estimates for older adults from different settings, they did not perform meta-regression to quantify it. Our study provided the evidence regarding how much the risk of prevalence increased for older adults who were hospitalized or residing in a nursing home compared with their community-dwelling counterparts. Our findings suggest that hospitalized older adults and those living in nursing homes are particularly vulnerable to this muscle disorder. Therefore, these populations should be prioritized in sarcopenia-screening and early-prevention efforts. Furthermore, intervention studies of sarcopenia targeting hospitalized older adults and those in nursing homes are warranted to inform evidence-based prevention and management efforts such as exercise and/or nutrition interventions for these susceptible groups. Meta-regression did not show significant difference in the prevalence estimate between the community-dwelling and outpatient groups for both genders. However, there were a small number of studies targeting outpatients, which might reduce the statistical power. Future research could further examine the risk of prevalent sarcopenia in this group of older adults.

We found the prevalence estimate in older men varied depending on the method used to assess muscle mass. Specifically, compared with the DXA method, the risk of prevalence was lower (OR: 0.58, 95% CI: 0.35 to 0.98) when using the BIA method. However, no such difference was detected in older women. There is some evidence that the BIA method tends to yield higher estimates of muscle mass, and therefore lower estimates of the prevalence of sarcopenia than the DXA method [91]. Wu and Li [24] synthesized the prevalence estimates from 16 original studies targeting community-dwelling Chinese older adults and obtained similar prevalence estimates irrespective of muscle mass assessments (DXA: 13%, BIA: 12%). However, they did not perform a meta-regression to further examine whether different muscle mass assessments could result in significantly different prevalence estimates after adjusting other covariates. In fact, the measurement of muscle mass remains a challenge in primary care settings; and, while DXA is the standard and more accurate method, BIA is preferred in primary care settings and research because it is more available, easier to use, and less expensive [92]. There are different types of equipment and frequencies of BIA. Based on previous study, BIA with a multifrequency device showed better agreement with the DXA method than that with other devices when assessing appendicular skeletal muscle mass [51]. The updated AWGS consensus in 2019 also recommended the use of multifrequency BIA [93]. However, the “model” of BIA measurement was not always stated explicitly in previous studies. Therefore, in future research, if the BIA method is used to assess muscle mass, the multifrequency type should be used and clearly documented.

Our study also found the risk of prevalent sarcopenia in older Chinese men living outside the mainland, specifically, in Hong Kong and Taiwan, to be nearly half that of their counterparts living on the mainland (OR: 0.47, 95% CI: 0.23 to 0.98). However, this difference was not significant in the case of older Chinese women. Regional variation in the prevalence of sarcopenia among community-dwelling Chinese older adults was also reported in a previous systematic review, with 17% in mainland and 6% in Hong Kong and Taiwan [24]. However, that review did not conduct meta-regression to quantify the regional variation in the prevalence of sarcopenia. Possible explanations for the variation between mainland Chinese and those living outside the mainland might be attributed to the different levels of economic development, different healthcare and dietary regimes, etc. [94]. Future studies could further explore the reasons why the prevalence of sarcopenia among older adults living outside the mainland is lower than for those in mainland China. Furthermore, as mainland China has a huge area with diverse geographic and climate variations, the prevalence of sarcopenia might also vary across different geographic locations. Until now, no study has investigated the epidemiological characteristics of sarcopenia among different geographic areas within China. Therefore, we further divided the area into 4 regions (south and east of mainland, west of mainland, north of mainland, and outside of mainland) and ran the univariable mate-regression. Findings showed that significant difference in the prevalence of sarcopenia was only presented between mainland areas and outside of the mainland; there was no difference among different geographic locations within mainland China in both genders (Supplementary Materials, Table S3). Therefore, we only divided the area into two regions (mainland and outside of the mainland) in the main analysis.

Previous observational studies found that older age was significantly associated with sarcopenia and severe sarcopenia in Chinese older adults [46,50]. However, less than half of included studies reported the gender-specific age (Supplementary Materials, Table S4). Therefore, our main results did not consider the age in the multivariable meta-regression. Instead, we conducted a supplementary analysis to fit the univariable meta-regression for age (details are presented in Supplementary Materials, Table S5). Findings indicated that the age group of 80 years and over showed a higher prevalence of sarcopenia compared with that of 60 to 70 years, which is consistent with current knowledge that the advanced age is an important risk factor of sarcopenia [50]. Body mass index (BMI) was also reported to be associated with the prevalence of sarcopenia in some literature, with a higher BMI relating to the lower prevalence of sarcopenia [46,50]. In this systematic review, BMI values reported in articles were all above 23 kg/m^2^ for both genders (Supplementary Materials, Table S4). We did not include BMI in the meta-regression, considering that the comparison between overweight and obesity might be less meaningful. For other possible factors which might influence the variance of prevalence reported, such as rural-urban regions, comorbidities, and others (e.g., type of dynamometer, protocol of gait speed test), due to limited information reported in the articles, we could not analyze them through meta-regression.

The present study was subject to several limitations. Firstly, a few of the included studies reported the prevalence by different age groups, so we could not pool the prevalence rate by age subgroup. However, age is a well-established factor of sarcopenia. The lower bound of age for participants in the studies included in this review ranged considerably, from 60 to 80 years. Therefore, age may be a significant source of the substantial heterogeneity that we found across studies. Future epidemiological research of sarcopenia is encouraged to report age-specific prevalence. Secondly, due to the small number of studies which reported the prevalence of sarcopenia for outpatients, we were unable to obtain a pooled prevalence rate for this group. Thirdly, we did not include all of the possible covariates in our meta-regression analysis to investigate the source of considerable variations in sarcopenia prevalence. For example, all of the included studies used dynamometers to assess muscle strength, but we had insufficient information to investigate the potential influence of the type of dynamometer used on the prevalence rate. We were likewise unable to analyze the influence of different measurement protocols used for the gait speed tests due to insufficient information. Finally, our results should be interpreted with caution because most of the included studies had a moderate risk of bias and significant heterogeneity.

## 5. Conclusions

This meta-analysis and meta-regression provides a comprehensive synthesis of sarcopenia prevalence in current literature targeting Chinese older populations not only from communities, but also from clinical settings and nursing homes. This is, to our knowledge, the first study to report pooled prevalence in Chinese older populations from clinical settings and nursing homes, and to examine the impact of populations from different settings, diagnostic criteria, muscle mass assessments, areas and walk distance of gait speed test on the prevalence estimate of sarcopenia via meta-regression analysis. Despite the variations in prevalence estimate across studies, this study revealed a certain proportion of Chinese older adults suffering from sarcopenia, especially for those who were hospitalized and residing in nursing homes. Considering the accelerating pace of aging, efforts should be made to implement early screening and lifestyle interventions such as nutrition and physical activity promotion to prevent this increasingly widespread age-related geriatric syndrome, especially for vulnerable groups.

## Figures and Tables

**Table 1:** Characteristics of included studies (*n* = 58).

Study	Language	Region	Design	Sample Size			Diagnostic Criteria	Assessment				Prevalence *n* (%)			Risk of Bias
								Muscle Mass	Muscle Strength	Physical Performance					
				Total	Male	Female				Distance (m)	Gait Speed	Total	Male	Female	
**Community (*n* = 32)**															
Meng et al., 2014 [45]	English	Mainland	Cross-sectional	101	101	—	EWGSOP	DXA	Dynamometer	6	Usual	46 (45.7)	46 (45.7)	—	Moderate
Wu et al., 2014 [46]	English	Taiwan	Cross-sectional	549	285	264	EWGSOP	BIA	Dynamometer	5	—	39 (7.1)	11 (3.9)	28 (10.6)	Moderate
Zhang et al., 2014 [47]	Chinese	Mainland	Cross-sectional	116	—	—	EWGSOP	DXA	Dynamometer	6	Usual	48 (41.4)	—	—	Moderate
Meng et al., 2015 [35]	English	Taiwan	Cross-sectional	771	412	359	EWGSOP	DXA	Dynamometer	5	Usual	44 (5.7)	35 (8.4)	9 (2.6)	Moderate
Wang et al., 2015 [48]	English	Mainland	Cross-sectional	316	164	152	AWGS	BIA	Dynamometer	4	Usual	94 (29.4)	43 (26.2)	51 (33.6)	Moderate
Wen et al., 2015 [36]	English	Mainland	Cross-sectional	286	136	150	IWGS	DXA	Dynamometer	6	Usual	17 (5.9)	10 (7.4)	7 (4.7)	Moderate
							EWGSOP					1 (0.3)	1 (0.8)	—	
							AWGS					9 (3.1)	8 (5.9)	1 (0.7)	
Chan et al., 2016 [19]	English	HK	Cross-sectional	3957	1979	1878	AWGS	DXA	Dynamometer	6	Usual	290 (7.3)	185 (9.3)	105 (5.6)	Low
Han et al., 2016 [49]	English	Taiwan	Cross-sectional	878	402	476	EWGSOP	BIA	Dynamometer	7	Usual	29 (3.3)	27 (6.7)	2 (0.4)	Moderate
Han et al., 2016 [50]	English	Mainland	Cross-sectional	1069	467	602	AWGS	BIA	Dynamometer	4	Usual	99 (9.3)	30 (6.4)	69 (11.5)	Moderate
Huang et al., 2016 [20]	English	Taiwan	Cross-sectional	731	386	345	AWGS	DXA	Dynamometer	6	—	50 (6.8)	36 (9.3)	14 (4.1)	Low
Wang et al., 2016 [51]	English	Mainland	Cross-sectional	944	462	482	AWGS	BIA	Dynamometer	6	Usual	98 (10.4)	38 (8.2)	60 (12.5)	Moderate
Wang et al., 2016 [52]	English	Mainland	Cross-sectional	854	404	450	AWGS	BIA	Dynamometer	4	Usual	96 (11.2)	53 (13.1)	43 (9.6)	Low
Xia et al., 2016 [37]	Chinese	Mainland	Cross-sectional	683	239	444	AWGS	BIA	Dynamometer	4	—	137 (20.1)	41 (17.2)	96 (21.6)	Moderate
Fang et al., 2017 [53]	Chinese	Mainland	Cross-sectional	106	—	106	AWGS	DXA	Dynamometer	6	Usual	13 (12.2)	—	13 (12.2)	Moderate
Hai et al., 2017 [54]	English	Mainland	Cross-sectional	836	415	421	AWGS	BIA	Dynamometer	6	Usual	88 (10.5)	47 (11.3)	41 (9.7)	Moderate
Hua et al., 2017 [55]	Chinese	Mainland	Cross-sectional	300	168	132	AWGS	BIA	Dynamometer	6	Usual	54 (18.0)	38 (22.6)	16 (12.1)	Moderate
Meng et al., 2017 [56]	Chinese	Mainland	Cross-sectional	106	101	5	AWGS	BIA	Dynamometer	—	—	29 (27.4)	—	—	Moderate
Chu 2018 [57]	Chinese	Mainland	Cross-sectional	191	69	122	AWGS	BIA	Dynamometer	4	Maximal	28 (14.7)	8 (11.6)	20 (16.4)	Moderate
Wang et al., 2018 [23]	English	Mainland	Cross-sectional	865	427	438	AWGS	BIA	Dynamometer	6	Usual	71 (7.1)	28 (6.6)	33 (7.5)	Moderate
Yang et al., 2018 [58]	English	Mainland	Cross-sectional	384	160	224	EWGSOP	BIA	Dynamometer	4	Usual	45 (11.72)	17 (10.6)	28 (12.5)	Moderate
Zhang et al., 2018 [38]	Chinese	Mainland	Cross-sectional	1148	368	780	AWGS	BIA	Dynamometer	6	Usual	164 (14.3)	55 (14.9)	109 (14.0)	Low
Chen et al., 2019 [21]	English	Mainland	Prospective	691	304	387	AWGS	BIA	Dynamometer	4	—	55 (8.0)	—	—	Moderate
Du et al., 2019 [22]	English	Mainland	Cross-sectional	631	213	418	AWGS	BIA	Dynamometer	6	Usual	77 (12.2)	41 (19.2)	36 (8.6)	Moderate
Liu et al., 2019 [39]	Chinese	Mainland	Cross-sectional	1723	915	808	AWGS	BIA	Dynamometer	6	Usual	121 (7.0)	96 (10.5)	25 (3.1)	Moderate
Liu 2019 [59]	Chinese	Mainland	Cross-sectional	769	416	353	AWGS	BIA	Dynamometer	6	Usual	32 (4.16)	12 (2.9)	20 (5.7)	Moderate
Wang et al., 2019 [60]	English	Mainland	Cross-sectional	945	465	480	AWGS	BIA	Dynamometer	6	Usual	276 (29.2)	123 (26.5)	153 (55.4)	Moderate
Xu et al., 2019 [40]	English	Mainland	Cross-sectional	2412	1012	1400	AWGS	BIA	Dynamometer	6	Usual	156 (6.5)	58 (5.7)	98 (7.0)	Moderate
Zhang et al., 2019 [61]	English	Mainland	Cross-sectional	1002	420	582	AWGS	BIA	Dynamometer	4	—	107 (10.7)	37 (8.8)	70 (12.0)	Moderate
Liu et al., 2020 [15]	English	Mainland	Cross-sectional	1712	—	—	AWGS	BIA	Dynamometer	4	Usual	556 (32.5)	—	—	Moderate
Rong et al., 2020 [62]	English	Mainland	Cross-sectional	450	266	184	AWGS	BIA	Dynamometer	6	Usual	89 (19.7)	50 (18.8)	39 (21.2)	Moderate
Xu et al., 2020 [63]	English	Mainland	Cross-sectional	582	246	336	AWGS	BIA	Dynamometer	6	Usual	15 (526.6)	82 (33.3)	73 (21.7)	Moderate
Yang et al., 2020 [64]	English	Mainland	Cross-sectional	483	184	299	FNIH	BIA	Dynamometer	4	Usual	16 (3.3)	11 (6.0)	5 (1.7)	Moderate
							IWGS					78 (16.1)	45 (24.5)	33 (11.0)	
							AWGS					44 (9.1)	20 (10.9)	24 (8.0)	
							EWGSOP1					76 (15.7)	41 (22.3)	35 (11.7)	
							EWGSOP2					22 (4.6)	12 (6.5)	10 (3.3)	
**Hospitals (*n* = 11)**															
Wang et al., 2016 [52] ^†^	English	Mainland	Cross-sectional	236	116	120	AWGS	BIA	Dynamometer	4	Usual	35 (14.8)	20 (17.2)	15 (12.5)	Low
Cui 2018 [65]	Chinese	Mainland	Cross-sectional	132	59	73	AWGS	DXA	Dynamometer	6	Usual	38 (28.8)	21 (35.6)	17 (23.3)	Moderate
Zhai et al., 2018 [66]	English	Mainland	Cross-sectional	494	216	278	AWGS	DXA	Dynamometer	6	—	158 (32.0)	87 (40.3)	71 (25.5)	Moderate
Chen et al., 2019 [67]	English	Mainland	Cross-sectional	118	92	26	AWGS	DXA	Dynamometer	6	Usual	71 (60.17)	65 (70.65)	6 (23.08)	Moderate
Wang 2019 [41]	Chinese	Mainland	Cross-sectional	119	64	55	AWGS	BIA	Dynamometer	—	—	26 (21.8)	17 (26.6)	9 (16.3)	Moderate
Yao 2019 [68]	Chinese	Mainland	Cross-sectional	378	153	225	AWGS	BIA	Dynamometer	6	Usual	47 (12.4)	15 (9.8)	32 (14.2)	Moderate
Yi et al., 2019 [69]	Chinese	Mainland	Cross-sectional	200	—	—	AWGS	BIA	Dynamometer	6	—	98 (49)	—	—	Moderate
Tan 2019 [70]	Chinese	Mainland	Cross-sectional	734	—	—	AWGS	BIA	Dynamometer	4	—	258 (35.1)	—	—	Moderate
Zhang et al., 2019 [71]	English	Mainland	Prospective	345	208	137	AWGS	BIA	Dynamometer	6	—	78 (22.6)	32 (15.4)	46 (33.6)	Moderate
Cui et al., 2020 [72]	English	Mainland	Cross-sectional	132	59	73	AWGS	DXA	Dynamometer	6	Usual	38 (28.8)	21 (55.3)	17 (44.7)	Moderate
Wang et al., 2020 [73]	Chinese	Mainland	Cross-sectional	236	144	92	AWGS	BIA	Dynamometer	6	—	63 (26.7)	28 (19.4)	35 (38.0)	Moderate
**Outpatient Services (*n* = 4)**															
Li et al., 2014 [74]	Chinese	Mainland	Cross-sectional	169	169	—	IWGS	DXA	Dynamometer	6	Usual	106 (62.9)	106 (62.9)	—	Moderate
							EWGSOP				Usual	56 (33.3)	56 (33.3)	—	
Wang et al., 2016 [75]	Chinese	Mainland	Cross-sectional	410	—	—	EWGSOP	DXA	Dynamometer	6	Usual	80 (19.5)	—	—	Moderate
Fung et al., 2019 [42]	English	Singapore	Cross-sectional	266	—	—	AWGS	BIA	Dynamometer	6	Usual	70 (26.3)	—	—	low
Wang et al., 2019 [76]	Chinese	Mainland	Cross-sectional	430	191	239	EWGSOP	BIA	Dynamometer	6	Usual	95 (22.1)	32 (16.8)	63 (26.4)	Moderate
**Nursing Home (*n* = 5)**															
Hsu et al., 2014 [77]	English	Taiwan	Cross-sectional	353	353	—	EWGSOP	BIA	Dynamometer	6	Usual	109 (30.9)	109 (30.9)	—	Moderate
Wu et al., 2017 [43]	Chinese	Mainland	Cross-sectional	786	320	466	EWGSOP	BIA	Dynamometer	4	—	199 (25.3)	64 (20.0)	135 (29.0)	Moderate
Liao 2018 [78]	Chinese	Mainland	Cross-sectional	225	63	162	AWGS	BIA	Dynamometer	6	Usual	86 (38.2)	26 (41.3)	60 (37.0)	Moderate
Zeng et al., 2018 [79]	English	Mainland	Cross-sectional	277	83	194	FNIH	BIA	Dynamometer	4	Usual	87 (31.4)	19 (22.9)	68 (35.1)	Moderate
Yang et al., 2019 [80]	English	Mainland	Cross-sectional	316	112	204	AWGS	BIA	Dynamometer	4	—	91 (28.8)	34 (30.4)	57 (27.9)	Moderate
**Mixed Settings: Communities and Nursing Homes (*n* = 2)**															
Chen 2018 [81]	Chinese	Mainland	Cross-sectional	158	43	115	AWGS	BIA	Dynamometer	6	Usual	34 (21.5)	5 (11.4)	29 (25.4)	Moderate
Yang 2018 [44]	Chinese	Mainland	Cross-sectional	316	112	204	AWGS	BIA	Dynamometer	4	Usual	91 (28.8)	34 (30.4)	57 (27.9)	Low
**Mixed Settings: Hospital and Outpatient Services (*n* = 5)**															
Feng 2016 [82]	Chinese	Mainland	Cross-sectional	330	157	173	AWGS	BIA	Dynamometer	4	Maximal	35 (10.6)	21 (13.4)	14 (8.1)	Moderate
Ma 2017 [83]	Chinese	Mainland	Cross-sectional	764	550	214	AWGS	BIA	Dynamometer	4	Usual	138 (18.1)	82 (14.9)	56 (26.2)	Moderate
Zhou et al., 2018 [84]	Chinese	Mainland	Cross-sectional	163	100	63	IWGS	DXA	Dynamometer	3	Maximal	26 (16.0)	—	—	Moderate
Zhang et al., 2019 [85]	Chinese	Mainland	Cross-sectional	223	—	—	AWGS	BIA	Dynamometer	6	Usual	49 (22.0)	—	—	Moderate
Yang 2019 [86]	Chinese	Mainland	Cross-sectional	102	51	51	AWGS	BIA	Dynamometer	4	Maximal	17 (16.0)	—	—	Moderate

^†^: This study provided sarcopenia prevalence for older adults from communities and clinical settings separately.

**Table 2:** Multivariable meta-regression.

Covariates	Males (*n* = 43)			Females (*n* = 41)		
	Exp (*β*)	95% CI	*p*-Value	Exp (*β*)	95% CI	*p*-Value
Populations						
Community-dwelling (ref)	1.00			1.00		
Outpatients	1.29	(0.52, 3.17)	0.570	2.28	(0.67, 7.73)	0.180
Hospitalized people	1.69	(1.01, 2.86)	0.047	2.10	(1.17, 3.78)	0.015
Nursing-home residents	2.50	(1.35, 4.66)	0.005	2.73	(1.38, 5.38)	0.005
Diagnosis criteria						
AWGS (ref)	1.00			1.00		
EWGSOP	1.23	(0.67, 2.27)	0.490	0.92	(0.39, 2.15)	0.840
Assessment of muscle mass						
DXA (ref)	1.00			1.00		
BIA	0.58	(0.35, 0.98)	0.044	1.17	(0.60, 2.29)	0.640
Area						
Mainland (ref)	1.00			1.00		
Out of mainland	0.47	(0.22, 0.98)	0.045	0.51	(0.18, 1.43)	0.190
Walk distance						
6 m (ref)	1.00			1.00		
4 m	0.84	(0.53, 1.32)	0.440	1.12	(0.68, 1.83)	0.650
Others	0.81	(0.34, 1.93)	0.630	0.73	(0.24, 2.25)	0.580

## Data Availability

Data sharing not applicable.

## Supplementary Materials

The following are available online at https://www.mdpi.com/article/10.3390/nu13051441/s1, S1: Search strategy; Table S2 Critical Appraisal of Study Quality; Table S3 Univariable meta-regression for regions; Table S4 Age and BMI reported in studies; Table S5 Univariable meta-regression for age.
