# Supplementary material for: The Prevalence of Sarcopenia in Chinese Older Adults: Meta-Analysis and Meta-Regression
Source: Nutrients. 2021 Apr 24;13(5):1441. doi: 10.3390/nu13051441 (PMC8146971; doi:10.3390/nu13051441)
Supplement: Supplementary file 1 [file nutrients-13-01441-s001.zip › nutrients-1124102-supplementary_citation revised.pdf]

Supplementary Materials

S1: Search strategy:

PubMed：

#1 Search ("Epidemiology"[Mesh]) OR "Prevalence"[Mesh]

#2 Search "Sarcopenia"[Mesh]

#3 Search ("China"[Mesh]) OR Chinese

#4 Search (("Aged"[Mesh]) OR elderly) OR older

#5 #1 AND #2 AND #3 AND #4

Wanfang:

#1 Ti/KW: (肌肉减少症) or Ti/KW: (肌少症))

#2: All: (老年人) or All: (老年)

#3 All: (患病率) or All:(流行)

#4 All: (中国) or All:(中国人)

#5 #1 AND #2 AND #3 AND #4

Table S2 Critical Appraisal of Study Quality

Study	External validity				Internal validity						
	Representation of target population	Representation of sampling frame	Random selection of sample	Minimal non-response bias	Data collected directly from subjects	Suitable case definition	Validated measurement	Consistent mode of data collection	Suitable length of the shortest prevalence period	Right calculation of prevalence	Risk of bias
Meng et al., 2014 [45]	No	No	No	Yes	Yes	Yes	Yes	Yes	Yes	Yes	Moderate
Wu et al., 2014 [46]	No	No	No	Yes	Yes	Yes	Yes	Yes	Yes	Yes	Moderate
Zhang et al., 2014[47]	No	No	No	Yes	Yes	Yes	Yes	Yes	Yes	Yes	Moderate
Meng et al., 2015 [35]	No	No	Yes	No	Yes	Yes	No	Yes	Yes	Yes	Moderate
Wang et al., 2015 [48]	No	No	No	Yes	Yes	Yes	No	Yes	Yes	Yes	Moderate
Wen et al., 2015 [36]	No	Yes	Yes	Yes	Yes	Yes	Yes	Yes	Yes	Yes	Low
Chan et al., 2016 [19]	No	Yes	Yes	Yes	Yes	Yes	Yes	Yes	Yes	Yes	Low
Han et al., 2016 [49]	No	No	No	Yes	Yes	Yes	Yes	Yes	Yes	Yes	Moderate
Han et al., 2016 [50]	No	No	No	Yes	Yes	Yes	Yes	Yes	Yes	Yes	Moderate
Huang et al., 2016 [20]	No	Yes	Yes	Yes	Yes	Yes	Yes	Yes	Yes	Yes	Low
Wang et al., 2016 [51]	No	No	No	Yes	Yes	Yes	Yes	Yes	Yes	Yes	Moderate
Wang et al., 2016 [52]	No	No	No	Yes	Yes	Yes	Yes	Yes	Yes	Yes	Moderate
Xia et al., 2016 [37]	No	No	Yes	Yes	Yes	Yes	No	Yes	Yes	Yes	Moderate
Fang et al., 2017 [53]	No	No	No	Yes	Yes	Yes	Yes	Yes	Yes	Yes	Moderate
Hai et al., 2017 [54]	No	No	No	Yes	Yes	Yes	Yes	Yes	Yes	Yes	Moderate
Hua et al., 2017 [55]	No	No	No	Yes	Yes	Yes	No	Yes	Yes	Yes	Moderate
Meng et al., 2017 [56]	No	No	No	Yes	Yes	Yes	No	Yes	Yes	Yes	Moderate

Chu 2018 [57]	No	No	No	Yes	Yes	Yes	Yes	Yes	Yes	Yes	Moderate
Wang et al., 2018 [23]	No	No	No	Yes	Yes	Yes	Yes	Yes	Yes	Yes	Moderate
Yang et al., 2018 [58]	No	No	No	Yes	Yes	Yes	Yes	Yes	Yes	Yes	Moderate
Zhang et al., 2018 [38]	No	Yes	Yes	Yes	Yes	Yes	Yes	Yes	Yes	Yes	Low
Chen et al., 2019 [21]	No	No	No	Yes	Yes	Yes	No	Yes	Yes	Yes	Moderate
Du et al., 2019 [22]	No	No	No	Yes	Yes	Yes	Yes	Yes	Yes	Yes	Moderate
Liu et al., 2019 [39]	No	No	Yes	Yes	Yes	Yes	Yes	Yes	Yes	Yes	Moderate
Liu 2019 [59]	No	No	No	Yes	Yes	Yes	Yes	Yes	Yes	Yes	Moderate
Wang et al., 2019 [60]	No	No	No	Yes	Yes	Yes	Yes	Yes	Yes	Yes	Moderate
Xu et al., 2019 [40]	No	Yes	Yes	Yes	Yes	Yes	Yes	Yes	Yes	Yes	Low
Zhang et al., 2019 [61]	No	No	No	Yes	Yes	Yes	No	Yes	Yes	Yes	Moderate
Liu et al., 2020 [15]	No	No	No	Yes	Yes	Yes	Yes	Yes	Yes	Yes	Moderate
Rong et al., 2020 [62]	No	No	No	Yes	Yes	Yes	Yes	Yes	Yes	Yes	Moderate
Xu et al., 2020 [63]	No	No	No	Yes	Yes	Yes	Yes	Yes	Yes	Yes	Moderate
Yang et al., 2020 [64]	No	No	No	Yes	Yes	Yes	Yes	Yes	Yes	Yes	Moderate
Cui 2018 [65]	No	No	No	Yes	Yes	Yes	Yes	Yes	Yes	Yes	Moderate
Zhai et al., 2018 [66]	No	No	No	Yes	Yes	Yes	Yes	Yes	Yes	Yes	Moderate
Chen et al., 2019 [67]	No	No	No	Yes	Yes	Yes	Yes	Yes	Yes	Yes	Moderate
Wang 2019 [41]	No	No	Yes	Yes	Yes	Yes	Yes	Yes	Yes	**Yes**	Moderate
Yao 2019 [68]	No	No	No	Yes	Yes	Yes	No	Yes	Yes	Yes	Moderate
Yi et al., 2019 [69]	No	No	No	Yes	Yes	Yes	Yes	Yes	Yes	Yes	Moderate
Tan 2019 [70]	No	No	No	Yes	Yes	Yes	Yes	Yes	Yes	Yes	Moderate
Zhang et al., 2019 [71]	No	No	No	Yes	Yes	Yes	Yes	Yes	Yes	Yes	Moderate
Cui et al., 2020 [72]	No	No	No	Yes	Yes	Yes	Yes	Yes	Yes	Yes	Moderate
Wang et al., 2020 [73]	No	No	No	Yes	Yes	Yes	Yes	Yes	Yes	Yes	Moderate
Li et al., 2014 [74]	No	No	No	Yes	Yes	Yes	Yes	Yes	Yes	Yes	Moderate

Wang et al., 2016 [75]	No	No	No	Yes	Yes	Yes	Yes	Yes	Yes	Yes	Moderate
Fung et al., 2019 [42]	No	Yes	Yes	Yes	Yes	Yes	Yes	Yes	Yes	Yes	Low
Wang et al., 2019 [76]	No	No	No	Yes	Yes	Yes	No	Yes	Yes	Yes	Moderate
Hsu et al., 2014 [77]	No	No	No	Yes	Yes	Yes	Yes	Yes	Yes	Yes	Moderate
Wu et al., 2017 [43]	No	Yes	Yes	Yes	Yes	Yes	No	Yes	Yes	Yes	Moderate
Liao 2018 [78]	No	No	No	Yes	Yes	Yes	Yes	Yes	Yes	Yes	Moderate
Zeng et al., 2018 [79]	No	No	No	Yes	Yes	Yes	Yes	Yes	Yes	Yes	Moderate
Yang et al., 2019 [80]	No	No	No	Yes	Yes	Yes	Yes	Yes	Yes	Yes	Moderate
Chen 2018 [81]	No	No	No	Yes	Yes	Yes	Yes	Yes	Yes	Yes	Moderate
Yang 2018 [44]	No	Yes	Yes	Yes	Yes	Yes	Yes	Yes	Yes	Yes	Low
Feng 2016 [82]	No	No	No	Yes	Yes	Yes	Yes	Yes	Yes	Yes	Moderate
Ma 2017 [83]	No	No	No	Yes	Yes	Yes	Yes	Yes	Yes	Yes	Moderate
Zhou et al., 2018 [84]	No	No	No	Yes	Yes	Yes	Yes	Yes	Yes	Yes	Moderate
Zhang et al., 2019 [85]	No	No	No	Yes	Yes	Yes	Yes	Yes	Yes	Yes	Moderate
Yang 2019 [86]	No	No	No	Yes	Yes	Yes	No	Yes	Yes	Yes	Moderate

Note: No=high risk; Yes=low risk.

Table S3 Univariable meta-regression for regions

Areas	Male (n=43)			Female (n=41)		
	**exp(β)**	**95% CI**	**P value**	**exp(β)**	**95% CI**	**P value**
North of mainland	1			1		
South and east of mainland	0.70	(0.398, 1.245)	0.220	0.60	(0.330, 1.092)	0.092
West of mainland	0.72	(0.385, 1.337)	0.287	0.70	(0.365, 1.330	0.265
Outside mainland	**0.43**	(0.207, 0.893)	0.025	**0.18**	(0.079, 0.417)	<0.001

Table S4 Age and BMI reported in studies

**Studies**	**Age***	**BMI***

	Total （n=21)	male （n=19）	Female （n=20）	Total (n-9)	male (n=13)	Female (n=14)
**Community (n=32)**	—	—	—	—	—	—
Meng et al., 2014 [45]	—	—	—	23.7	—	—
Wu et al., 2014 [46]	76.0	—	—	24.6	—	—
Zhang et al., 2014[47]	88.2	—	—	24.9	—	—
Meng et al., 2015 [35]	—	74.8	73.0	—	—	—
Wang et al., 2015 [48]	—	75.6	76.9	—	23.1	23.6
Wen et al., 2015 [36]	—	66.5	65.4	—	23.4	23.1
Chan et al., 2016 [19]	—	72.4	72.6	—	23.4	23.9
Han et al., 2016 [49]	72.7	73.1	71.9	23.8	24.1	23.6
Han et al., 2016 [50]	67.3	68.0	66.7	—	25.2	25.4
Huang et al., 2016 [20]	73.4	74.1	72.6	—	—	—
Wang et al., 2016 [51]	68.7	69.1	68.5	23.9	23.7	24.0
Wang et al., 2016 [52]	—	69.9	68.8	—	23.8	23.8
Xia et al., 2016 [37]	68.7	—	—	—	—	—
Fang et al., 2017 [53]	—	—	69.9	—	—	23.5
Hai et al., 2017 [54]	68.6	68.9	68.3	—	23.9	24.1

Hua 2017 et al., [55]	—	—	—	—	—	—
Meng et al., 2017 [56]	—	—	—	—	—	—
Chu 2018 [57]	68.6	—	—	23.7	—	—
Wang et al., 2018 [23]	—	—	—	—	—	—
Yang et al., 2018 [58]	71.5	71.5	71.0	—	24.1	24.2
Zhang et al., 2018 [38]	—	—	—	—	—	—
Chen et al., 2019 [21]	67.5	—	—	—	—	—
Du et al., 2019 [22]	—	—	—	—	—	—
Liu et al., 2019 [39]	—	—	—	—	—	—
Liu 2019 [59]	—	—	—	—	—	—
Wang et al., 2019 [60]	68.8	69.1	68.4	—	—	—
Xu et al., 2019 [40]	—	68.6	68.1	—	24.3	25.3
Zhang et al., 2019 [61]	—	—	72.3	—	—	—
Liu et al., 2020 [15]	—	—	—	—	—	—
Rong et al., 2020 [62]	—	—	—	—	—	—
Xu et al., 2020 [63]	86.4	—	—	—	—	—
Yang et al., 2020 [64]	66.8	66.3	67.1	24.9	25.1	24.7
**Hospitals (n=11)**				—		
Wang et al., 2016 [52] †		68.4	68.9	—	24.4	24.1
Cui 2018 [65]	71.3	—	—	—	—	—
Zhai et al., 2018 [66]	84.2	—	—	—	—	—
Chen et al., 2019 [67]	—	—	—	—	—	—

Wang 2019 [41]	—	—	—	—	—	—
Yao 2019 [68]	—	—	—	—	—	—
Yi et al., 2019 [69]	—	—	—	—	—	—
Tan 2019 [70]	—	—	—	—	—	—
Zhang et al., 2019 [71]	—	—	—	24.9	—	—
Cui et al., 2020 [72]	—	—	—	—	—	—
Wang et al., 2020 [73]	—	—	—	—	—	—
**Outpatient services(n=4)**	—	—	—	—	—	—
Li et al., 2014 [74]	—	—	—	—	—	—
Wang et al., 2016 [75]	77.6	—	—	—	—	—
Fung et al., 2019 [42]	68.3	—	—	—	—	—
Wang et al., 2019 [76]	—	—	—	—	—	—
**Nursing home (n=5)**	—	—	—	—	—	—
Hsu et al., 2014 [77]	—	82.7	—	—	—	—
Wu et al., 2017 [43]	—	79.1	85.7	—	—	—
Liao 2018 [78]	—	85.0	83.2	—	—	—
Zeng et al., 2018 [79]	81.6	80.7	82.0	24.2	24.2	24.1
Yang et al., 2019 [80]	—	—	—	—	—	—
**Mixed settings: communities and nursing homes (n=2)**						
Chen 2018 [81]	—	—	—	—	—	—
Yang 2018 [44]	—	—	—	—	—	—
**Mixed settings: hospital and outpatient services (n=5)**						
Feng 2016 [82]	—	—	—	—	—	—
Ma 2017 [83]	74.0	—	—	—	—	—
Zhou et al., 2018 [84]	78.0	—	—	—	—	—

Zhang et al., 2019 [85]	—	—	—	—	—	—
Yang 2019 [86]	—	—	—	—	—	—

*: mean values

†: This study provided sarcopenia prevalence for older adults from communities and clinical settings, separately.

Table S5 Univariable meta-regression for age

Variables	Male (n=19)			Female (n=20)		
	**Exp(β)**	**95% CI**	**P value**	**Exp(β)**	**95% CI**	**P value**
**Age**						
60-70 years	1			1		
70-80 years	1.11	(0.641, 1.910)	0.700	0.65	(0.270, 1.554)	0.309
80 years above	**2.66**	(1.284, 5.497)	0.012	**3.28**	(1.089, 9.907)	0.036
